# Epidemiology and pathology of Paget’s disease of bone – a review

**DOI:** 10.1007/s10354-016-0496-4

**Published:** 2016-09-06

**Authors:** Elena Nebot Valenzuela, Peter Pietschmann

**Affiliations:** 1 Department of Pathophysiology and Allergy Research, Center of Pathophysiology, Infectiology and Immunology, Medical University of Vienna, Waehringer Guertel 18–20, 1090 Vienna, Austria; 2 Department of Physiology, School of Pharmacy, and Institute of Nutrition and Food Technology, University of Granada, Granada, Spain

**Keywords:** Pagetic bone, Bone structure, Histology, Histomorphometry, Skeletal disorder, Morbus Paget, Knochenstruktur, Histologie, Histomorphometrie, Knochenerkrankung

## Abstract

Paget’s disease of bone (PDB) is a noninflammatory, metabolic, skeletal disorder characterized by localized excessive osteoclastic bone resorption that is followed by compensatory increased osteoblastic activity leading to unstructured, fibroblastic, and biomechanically unstable bone. As a result, there is deformity and enlargement of the bone with a defective and disorganized pattern. Here, we review the epidemiology, etiology, pathology, macrostructure, histology, and quantitative histomorphometry findings of PDB. Hyperosteoclastosis and poor definition of the boundary between cortical and medullary bone are the main histological findings in PDB. Additionally, Pagetic bone is also characterized by hypertrophy and alteration of trabecular parameters.

## Zusammenfassung

Der Morbus Paget ist eine nichtentzündliche metabolische Knochenerkrankung, die durch eine lokale, übermäßige Knochenresorption mit kompensatorischer Steigerung der Osteoblastenaktivität gekennzeichnet ist. In Folge kommt es zu einem veränderten, fibrösen und biomechanisch instabilen Knochen sowie zu Deformierungen und Verdickungen des Knochens mit einer gestörten und desorganisierten Struktur. In diesem Beitrag geben wir eine Übersicht über die Epidemiologie, Ätiologie, Pathologie, Makrostruktur, Histologie und die quantitative Histomorphometrie des Morbus Paget. Das Auftreten von Riesenosteoklasten und die schlechte Abgrenzbarkeit von kortikalem und trabekulärem Knochen sind wichtige histologische Kennzeichen der Erkrankung. Darüber hinaus ist der Knochen bei Morbus Paget auch durch eine Hypertrophie und Veränderungen der Trabekelstruktur gekennzeichnet.

## Introduction

Paget’s disease of bone (PDB) was originally described in a report that has become a classic in the medical literature. James Paget called the disease *osteitis deformans*, in part because of the extensive and deforming changes that took place in the skeleton in severe cases [1]. The disease is a chronic bone abnormality, which may affect a single, several, or many bones, but never involves the entire skeleton. The cause remains unknown. Nevertheless, a prevalent hypothesis is that the disease is initiated by a slow virus in a genetically vulnerable patient [2], because nuclear inclusions of viral components have been observed in osteoclasts from affected patients [3].

**Fig. 1:**
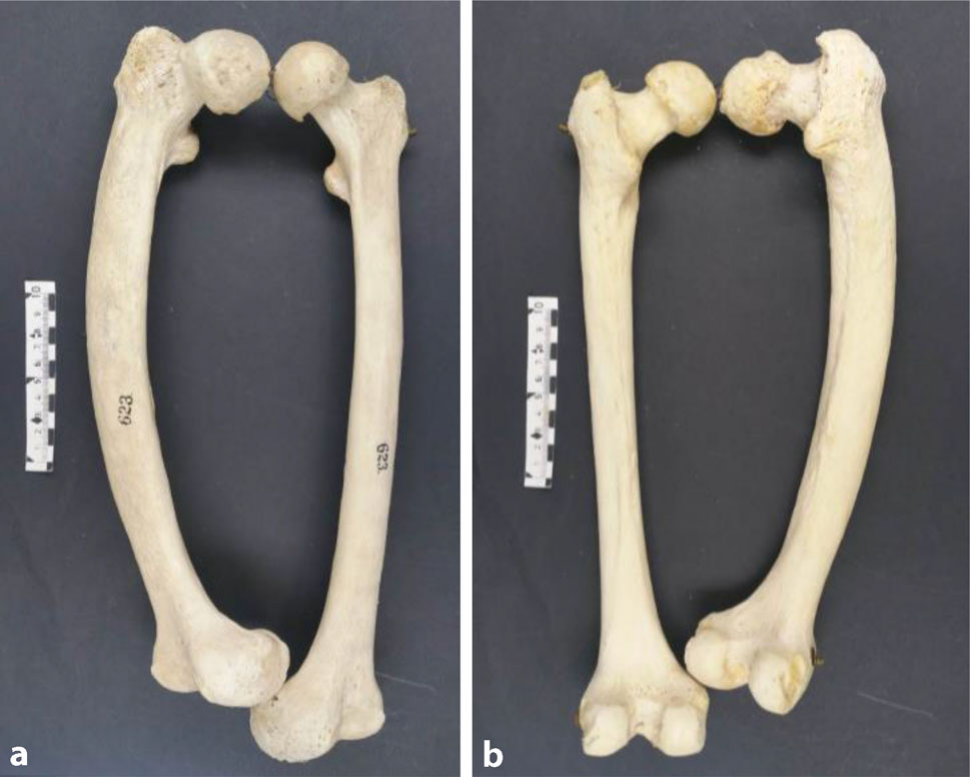
Pagetic human femur, unknown gender and age, compared to the healthy femur of the same individual. **a** Anterior view, **b** posterior view. The bones were obtained from the Pathologic-Anatomical Collection in The Fools Tower, Museum of Natural History, Vienna, Austria

**Fig. 2:**
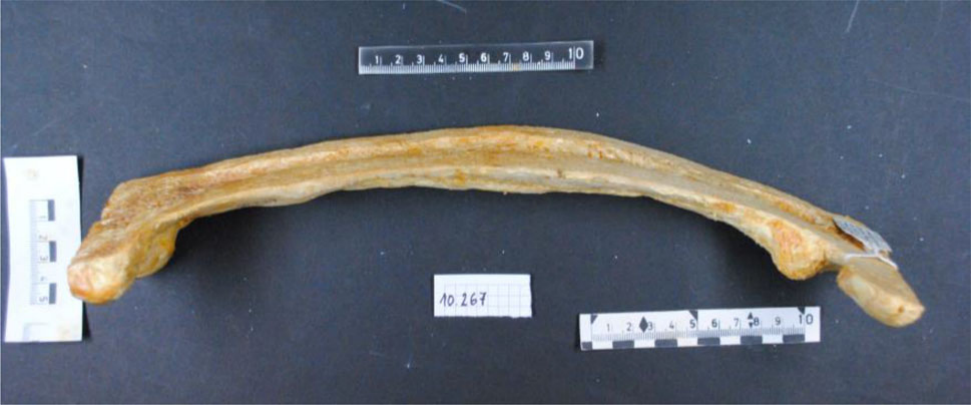
Lateral view of a Pagetic human femur, unknown gender and age. The bone was obtained from the Pathologic-Anatomical Collection in The Fools Tower, Museum of Natural History, Vienna, Austria

Paget’s disease of bone is the paradigm of a focal bone disorder with accelerated bone turnover [4]. It is a noninflammatory, metabolic, skeletal disorder characterized by localized excessive osteoclastic bone resorption that is followed by compensatory increased osteoblastic activity [5] leading to unstructured, fibroblastic, and biomechanically unstable bone [6]. As a result, there is deformity and enlargement of the bone with a defective and disorganized pattern (plexiform bone) (Fig. 1 and 2); therefore, Pagetic bone is susceptible to fractures [4]. The axial skeleton is frequently involved and the bones most commonly affected include the pelvis (70 %), femur (55 %), lumbar spine (53 %), skull (42 %), and tibia (32 %) [7, 8]. Nevertheless, Pagetic bone lesions can occur at any site of the skeleton [6].

## Epidemiology

The diagnosis of PDB is rare before age 50. The disease affects both men and women [9]; in most series males predominate. In 1932, Schmorl [10] found a prevalence of 3 % of PDB in a series of over 4600 autopsies of individuals above 40 years of age.

Its geographical distribution is uneven, with areas of high prevalence with familial aggregation detected in most series [4]. Paget’s disease occurs most commonly in people of British descent. The disease is also common in British migrants to countries like Australia, New Zealand, and North America, as well as in other countries in Europe, such as in France, Germany, Spain, or Italy [11]. Spain is considered to have a medium–low prevalence compared to other European countries, approximately 0.9–1.3 % of the population over 65 years [12]. The study by Poór et al. [13] described the frequency of the disorder in eight European cities, showing the lowest prevalence rate among hospital patients ≥55 years old in the Austrian city of Innsbruck (0.2 %).

A study by Van Staa et al. [14] evaluated the age- and gender-specific incidence of PDB in England and Wales in the adult population. They concluded that the disorder was more frequent among men of all ages over 55 years. The incidence increased steeply with age among both men and women, and was estimated at 0.3 cases per 10,000 person-years among women aged 55–59 years and 0.5 cases per 10,000 person-years among men of similar age. At the age of ≥85 years, this rate rose to 5.4 among women and 7.6 among men. Based on these assumptions, the prevalence of clinically diagnosed PDB is 0.3 % among men and women ≥55 years old.

There is evidence that PDB has become less common and less severe over the past quarter of a century in the UK and many other countries [13]. The decrease in the incidence of canine distemper or measles virus infection due to the introduction of vaccination in Europe may be associated with the decline of PDB [13]. Previous studies described PDB, after osteoporosis, as the second most common metabolic bone disease [10, 15].

## Etiology

Studies of patients with Paget’s disease indicate that there is a family history of the disorder in 5 [16] to 40 % [17]. There is an autosomal dominant transmission pattern [9]. Mutations in the gene-producing sequestosome 1 increase susceptibility to the development of Paget’s disease [18], but there is incomplete penetrance of the disease in some family members who have been found to harbor gene mutations [19]. Other genes have also been implicated in increasing susceptibility to develop the disorder [20], and nearly all of these genes, including the sequestosome 1 gene, are involved in osteoclast biology.

An additional role for sequestosome 1 is in autophagy. Sequestosome 1 has been shown to interact with an autophagic protein. Because of the presence of inclusion bodies found in the osteoclasts of Pagetic bones, dysregulation of the autophagy process may be part of the pathogenesis of PDB [21]. Recently, the study by McManus et al. [22] indicated a strong potential regulatory role for the kinase associated with the response to the receptor activator of NF-κB ligand (RANKL) activation in osteoclast stimulatory pathways and autophagy induction, which may contribute to the osteoclast phenotype in PDB.

Other investigations of the etiology of Paget’s disease have focused on the potential role of chronic paramyxovirus infections contributing to the pathogenesis of the disorder [23]. The most impressive animal model of Paget’s disease has been generated in transgenic mice by targeting the measles virus nucleocapsid protein and a mutated sequestosome 1 gene into the animals [24]. Immunocytochemical studies have shown that Pagetic osteoclasts contain paramyxoviral-like nuclear inclusions that cross-react with antibodies to measles virus, respiratory syncytial virus, and canine distemper virus nucleocapsid antigen [23, 25, 26]. Nevertheless, the issue of whether or not viral infections are related to PDB is not resolved [27].

Although the primary cause of these abnormalities in Paget’s osteoclasts is still unknown [28], osteoclasts are abundant in Paget’s lesions. They are also larger, contain increased nuclei per osteoclast, have increased bone resorbing capacity, increased 1,25-dihydroxyvitamin D3 (1,25-(OH)_2_D_3_) and RANKL responsivity, and secrete high levels of interleukin 6 (IL-6) compared to normal osteoclasts [29, 30]. The increase in osteoclast numbers can be explained in part by the high levels of expression of several factors in Paget’s osteoclasts which are directly related to osteoclast formation and activation, such as the *c-fos* protooncogene [31], IL-6, IL-6 receptor, and NF-κB [29]. In addition, Paget’s osteoclasts seem to respond differently to osteotropic factors such as calcitonin and 1,25-(OH)_2_D_3_ [32]. Pagetic osteoclasts frequently express the measles virus nucleocapsid protein [33], which induces high levels of IL-6 expression in both human and mouse osteoclasts, and results in the development of Pagetic bone lesions in mice in vivo [34].

## The Pagetic bone lesion

### Macrostructure

**Fig. 3:**
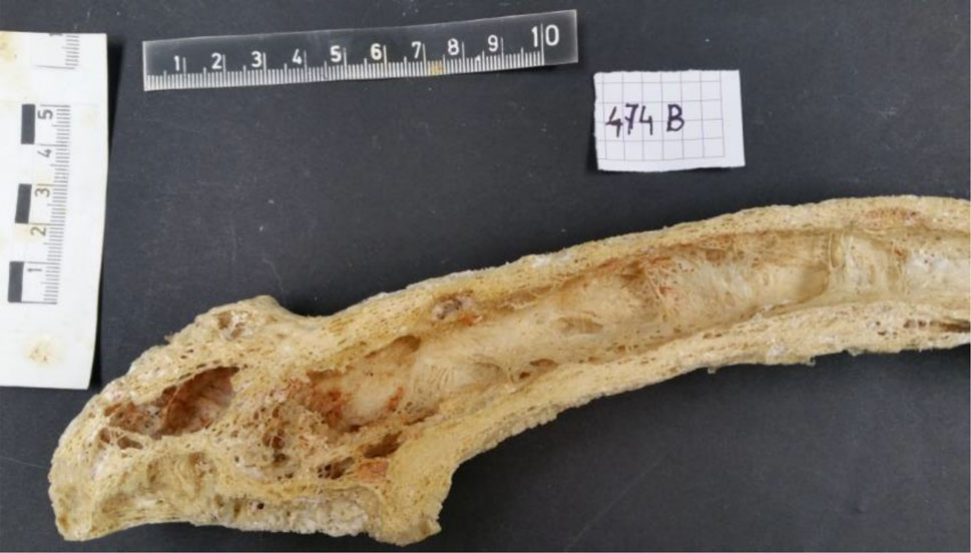
Inside view of a Pagetic human femur, female, 71 years old. The bone was obtained from the Pathologic-Anatomical Collection in The Fools Tower, Museum of Natural History, Vienna, Austria

Most patients are asymptomatic [4], whereas some develop complications such as bone pain, osteoarthritis, fracture, deformity, deafness, and nerve compression syndromes [6]. The early lesions are predominantly lytic and osteoporotic; bone resorption predominates with abnormally large osteoclasts containing multiple pleomorphic nuclei and microfilamentous inclusion bodies [35]. Later, a mixed osteolytic-osteoblastic phase with an abundance of osteoblasts forming new matrix in the form of woven bone [36] occurs, where thickening of the cortex by endosteal and periosteal bone deposition with enlargement of the bones is observed (Fig. 3). The trabecular architecture becomes accentuated and its usually smooth outline assumes irregular surface contours in radiographs [1]. Within the diaphyseal cortex, the primary resorption phase of PDB is often limited either to the endosteum or to the central layers of the cortex. This results in primary resorption fronts that are usually discrete, both radiologically and scintigraphically. The subsequent activation of the subperiosteal cortex may be delayed, leading to secondary expanding fronts associated with subperiosteal new bone formation [37]. This alternation of resorptive and sclerotic areas creates a mottled appearance on X‑ray films.

### Histology

A general description of histological findings in Pagetic bones is summarized in Table 1. Most of the studies on the histology of PDB have focused on iliac crest bone [6, 36, 38–40] or on vertebrae [41, 42]. As mentioned before, PDB has the primary cellular abnormality residing in osteoclasts [43], which are increased in number and size, and contain many more nuclei per cell compared to normal osteoclasts [44]. The histological study by Seitz et al. [6], using the Hamburger Bone Register, described that trabecular bone appeared mostly isolated, with a clumsy composition in Pagetic iliac crests biopsies. Multinucleated osteoclasts with more than 12 nuclei per cell were frequently detected at the trabecular bone surface. Moreover, the authors observed a typical appearance of deep resorption lacunae with the so-called swallowtail pattern. As a sign of accelerated bone formation, they also found an increase in osteoid surfaces and activated cuboidal osteoblasts, and described that collagen fibers were not oriented in one direction, but rather displayed a random distribution indicative of woven bone [6].

**Table 1:** Description of histological findings in Pagetic bones

Category	Description	Comment	Parameters	Direction	References
Osteoclasts and resorption of bone	Hyperosteoclastosis associated with fibrosis	Number, size, and nuclearity:10-times the number of osteoclasts; large: over 200 µm in diameter, around 150 nuclei per osteoclast (many pyknotic)	Osteoclasts number Osteoclasts surface	Increase	[6, 11, 36, 42, 60]
			Rate of bone resorption Erosion rate of bone	Increase	[36]
			Nuclear inclusions	Presence	[61, 62]
	Resorption surfaces	Extend irregularly in multiple directions and are unusually deep	Total resorption surfaces	Increase	[63]
			Osteoclastic lacunae	Irregular	[60]
Osteoblasts and deposition of bone	Chaotic fashion resulting in woven bone in typical “mosaic” which is mechanically weak	Poor definition of the boundary between cortical and medullary bone	–	–	[11, 36, 64]
	Hyperosteoblastosis associated with an extension of the osteoid borders	–	Osteoblast number Osteoblast surface Osteoblastic surfaces Trabecular osteoid volume Trabecular osteoid surfaces	Increase	[6, 36, 42]
	Alternating heavily calcified and fibrotic areas	Isolated areas of poorly mineralized osteoid	Fibrous tissue	Increase	[6, 36, 42, 64]
			Thickness index of the osteoid borders	Decrease	[36, 60]
	Osteoblastic appositional rate	–	Calcification rate (mineral apposition rate)	Increase	[36]
Hypervascularization	–	–	Vascularity and marrow fibrosis	Increase	[11, 36]

## Quantitative histomorphometry findings

Analyses of bone structure in Paget’s disease on a quantitative (histomorphometric) level are surprisingly rare. A histomorphometric study carried out in two medieval preparations with PDB found evidence of an increased trabecular thickness [45]. Histomorphometric results from Seitz et al. [6] showed a high bone turnover with a significant increase in bone resorption and bone formation indices (trabecular number, osteoid volume and osteoid surface, osteoblast number and surface of osteoclasts), and an increased bone volume. However, Lauffenburger et al. [38] stated that in PDB, there is a better correlation between bone formation and bone resorption than in osteoporosis. On the other hand, Cherian et al. [41] observed that bone density was increased in vertebrae affected by PDB, and the contribution from cortical and trabecular bone was in the ratio expected in normal bone. Cortical quantitative computed tomography values were underestimated in PDB compared with physical measurements of density. Furthermore, in the histomorphometric analysis of Petska et al. [42], affected vertebral body biopsies revealed a significant increase both in trabecular bone volume as well as in osteoid parameters. In comparison to histomorphometric data obtained from extraspinal skeletal locations affected by PDB (i. e., iliac crest), a similar bone microarchitecture of the vertebral bodies was observed. They concluded that vertebral body height and the spine bone volume together with bone density might play an important role in the manifestation of Pagetic bone alterations [42]. There is also a histopathology study based on temporal bone [46], in which authors only studied eight subjects; nevertheless, no quantitative results were shown. A general description of structural findings in Pagetic bones is summarized in Table 2.

**Table 2:** Description of structural findings in Pagetic bones

Category	Description/comment	Parameters	Direction	References
Bone architecture and lamellar texture	Small patches, scalloped contours and interlocked by polycyclic cement lines: “structure of a puzzle”	Periosteocytic lacunae size in the woven zones	Increase	[36]
Trabecular microarchitecture	Trabeculae are thick and numerous	Trabecular bone volume Trabecular number	Increase	Iliac crest [6] Spine [42]
		Density of the bone tissue	Increase	[60]
		Trabecular separation	Decrease	Iliac crest [6] Spine [42]
		Trabecular thickness	Not altered	Iliac crest [6] Spine [42]
Hypertrophy of the bones	Thickening and elongation of the bones	–	–	[36, 60]
Non-mineralized bone (osteoid)	–	Osteoid volume Osteoid surface Osteoblast surface relative to the osteoid surface	Increase	Iliac crest [6] Spine [42]

An understanding of the normal skeletal distribution and the architecture of cancellous bone seems to be essential for further knowledge of both the role of bone cellular activity and also the diagnostic interpretation of bone volume measurements [47]. There are striking differences between peripheral and axial measurement sites, and even between local areas of both. For instance, in normal subjects, trabecular bone volume at the femoral neck is higher than at the lumbar spine or the iliac crest. Of note, there is a systematic variation in trabecular microarchitecture of the iliac crest, showing the highest bone mass within the anterior part and lower values for the medial and dorsal parts [47].

The functional attribution is most remarkable for peripheral cancellous bone, such as in the metaphysis of the long bones [48]. Thus, the relationship between the trabecular bone mass at different skeletal sites has been the subject of several previous studies [49–52]. Although these studies helped to gain an insight into bone mass, bone structure, and bone diminution at different sites under different diseases, their results were conflicting [53–56]. An improvement of knowledge about pathological skeletal conditions will depend on a better understanding of the physiological distribution of trabecular bone throughout the skeleton [47]. Considering the fact that the structure of trabecular bone is complex and that considerable skeletal heterogeneity exists [50], studies of Pagetic bone structure in different regions of the skeleton are of major interest.

Nevertheless, there are no histomorphometric studies based on long bones, only a case report [57] of a femur fracture associated with PDB in an Asian patient. Long bones are commonly affected by Paget’s disease (55 % femur and 32 % tibia) [58], thus it is crucial to get pertinent information about the basis of skeletal complications including bowing deformities, fractures of the Pagetic bone, and osteosarcoma [58].

In addition to long bones, Paget’s disease affecting the skull is of particular clinical importance due to its proximity to the nervous system. Neurologic syndromes associated with Paget’s disease include headache, dementia, brainstem and cerebellar dysfunction, cranial neuropathies, myelopathy, cauda equina syndrome, and radiculopathies [59].

## Future research directions

Since several studies of Pagetic bone structure in different regions of the skeleton reveal similar findings, we put forward the hypothesis that—despite the phenomenon of skeletal heterogeneity—in PDB, bone microarchitecture is altered independent of the anatomic localization of the lesion in a uniform manner. Identification of microstructure of Pagetic bones and analyses of histological samples not only helps to clarify the pathogenesis of PDB, but may also contribute to a better knowledge about the physiological distribution of cortical and trabecular bone throughout the skeleton.

The scientific community needs further research on bone microarchitecture, skeletal distribution, and histological and histomorphometric characteristics in bone samples with Paget’s disease.
